
JOURNAL INFORMATION
==============================
NLM Title Abbreviation: Function (Oxf)
Journal ID: Function (Oxf)
NLM Journal ID: 101770668
Journal ID: function

Function (Oxford, England)

EISSN: 2633-8823

ARTICLE INFORMATION
==============================
PMCID: PMC7717325
PMID: 35321097
DOI: 10.1093/function/zqaa032
Article ID: zqaa032
Article version: 1
Subjects: Perspectives, AcademicSubjects/SCI00960, AcademicSubjects/MED00772, AcademicSubjects/SCI01270, AcademicSubjects/SCI01360

Is carotid body infection responsible for silent hypoxemia in COVID-19 patients?

Villadiego Javier
Ramírez-Lorca Reposo
Cala Fernando
Labandeira-García José L
Esteban Mariano
Toledo-Aral Juan J
López-Barneo José lbarneo@us.es
z1 Institute of Biomedicine of Seville (IBiS) and Department of Medical Physiology and Biophysics, Universidad de Sevilla/CSIC/Hospital Universitario Virgen del Rocío , Spain
z2 Networking Research Center on Neurodegenerative Diseases (CIBERNED) , Spain
z3 Research Center for Molecular Medicine and Chronic Diseases (CIMUS), Universidad de Santiago de Compostela , Spain
z4 Centro Nacional de Biotecnología, CSIC , Spain
Corresponding author: José López-Barneo MD & PhD, Instituto de Biomedicina de Sevilla (IBiS), Campus Hospital Universitario Virgen del Rocío, Avenida Manuel Siurot s/n, 41013 Sevilla, Spain, +34 609269027 or + 34 955923032 lbarneo@us.es
Electronic publication date: 2020 Nov 23
Electronic Location ID: zqaa032
Received 2020 Nov 17; Accepted 2020 Nov 18
Copyright: © The Author(s) (2020). Published by Oxford University Press on behalf of American Physiological Society.
Copyright year: 2020
License: This is an Open Access article distributed under the terms of the Creative Commons Attribution License (http://creativecommons.org/licenses/by/4.0/), which permits unrestricted reuse, distribution, and reproduction in any medium, provided the original work is properly cited.
License URL: https://creativecommons.org/licenses/by/4.0/

Page count: 8
edited-state: accepted-manuscript
article-lifecycle: PAP

==============================
FUNCTION™

american physiological society

### Is carotid body infection responsible for silent hypoxemia in COVID-19 patients?

Javier Villadiego[1,2], Reposo Ramírez-Lorca[1], Fernando Cala[1], José L. Labandeira-García[2,3], Mariano Esteban[4], Juan J. Toledo-Aral[1,2], and José López-Barneo[1,2]*

1. Institute of Biomedicine of Seville (IBiS) and Department of Medical Physiology and Biophysics, Universidad de Sevilla/CSIC/Hospital Universitario Virgen del Rocío, Spain
2. Networking Research Center on Neurodegenerative Diseases (CIBERNED), Spain
3. Research Center for Molecular Medicine and Chronic Diseases (CIMUS), Universidad de Santiago de Compostela, Spain
4. Centro Nacional de Biotecnología, CSIC, Spain

* Corresponding author:
José López-Barneo MD & PhD
Instituto de Biomedicina de Sevilla (IBiS)
Campus Hospital Universitario Virgen del Rocío
Avenida Manuel Siurot s/n
41013 Sevilla, Spain
+34 609269027 or +34 955923032
lbarneo@us.es

© The Author(s) 2020. Published by Oxford University Press on behalf of the American Physiological Society.

This is an Open Access article distributed under the terms of the Creative Commons Attribution License (http://creativecommons.org/licenses/by/4.0/), which permits unrestricted reuse, distribution, and reproduction in any medium, provided the original work is properly cited.

The pathogenic mechanisms underlying the symptomatology of coronavirus disease 2019 (COVID-19) patients are not well understood. An atypical and bewildering clinical manifestation found in many COVID-19 patients is that they exhibit severe hypoxemia, with arterial levels of oxygen ($O_2$) tension even below 50 mmHg, without clear signs of distress (dyspnea) or significant acceleration of breathing.[1,2] Under these conditions, patients with COVID-19 pneumonia may decompensate and as a consequence undergo a rapid deterioration of their clinical state that can eventually lead to death. The pathophysiology of this so-called “silent hypoxemia”[4] or “happy hypoxia” is unknown.[1,3,4] A decline in arterial $O_2$ tension is normally detected by $O_2$-sensing cells in the carotid body (CB), the main arterial chemoreceptor, which rapidly activate sensory fibers impinging on neurons in the brainstem to induce compensatory hyperventilation and increased heart rate. In this way, both $O_2$ uptake and its distribution to the tissues are enhanced. Bilateral removal of the CB in humans leaves individuals unaware of hypoxemia, with complete abolition of the hypoxic ventilatory response.[5] Therefore, inhibition of CB responsiveness to hypoxia could be a plausible explanation for the impaired respiratory drive and reduced dyspnea that characterize the “silent hypoxemia” observed in COVID-19 patients.

The CB parenchyma is organized into clusters of cells called glomeruli. Each glomerulus is composed of 4-8 neuron-like glomus, or type I, cells, which are in close contact with a network of fenestrated capillaries and are richly innervated by afferent sensory fibers of the petrosal ganglion. Glomus cells, the $O_2$-sensing elements in the CB, contain abundant synaptic vesicles with neurotransmitters that are rapidly released in response to hypoxia to activate the sensory fibers connecting with the brainstem respiratory and autonomic centers.[5] Acute responsiveness of glomus cells to hypoxia depends on a specialized mitochondrial $O_2$ sensing and signaling system, which is based on the expression of specific ion channels, mitochondrial enzymes, and subunits of the electron transport chain (ETC).[5,6] In addition to the chemosensitive glomus cells, which can be easily identified by antibodies against tyrosine hydroxylase (TH), the CB glomeruli also contain a smaller number of glial-like, type II or sustentacular cells with interdigitating processes that envelop the glomus cells. Type II cells are multipotent stem cells that can differentiate into $O_2$ sensitive glomus cells to support CB growth under sustained hypoxia.[5] Although acute $O_2$-sensing is an intrinsic property of CB glomus cells, the functional responses of these cells are https://mc.manuscriptcentral.com/function modulated by numerous auto- and paracrine signals generated within the organ. In this regard, a local renin-angiotensin system (RAS) and its principal components (angiotensinogen, angiotensin converting enzyme, and angiotensin receptors) have been described in the CB.[7] Given that angiotensin-converting enzyme 2 (ACE2) has an important regulatory role in the RAS and has been identified as the functional receptor by which severe acute respiratory syndrome coronavirus 2 (SARS-CoV-2) enters human cells,[8] we hypothesized that this enzyme could also be part of the CB local RAS and thus make CB cells a potential target for SARS-CoV-2 viral infection.

To move this idea forward, we studied the immunohistochemical expression of ACE2 in adult human CB tissue using thin histological sections obtained from cadaveric donors available in the tissue bank of our institution. We showed that CB tissue expresses significant levels of ACE2. As expected, ACE2 staining appeared in blood vessels (Figure 1A, v), as it is known that this enzyme is highly expressed in endothelial and vascular smooth muscle cells. However, strong ACE2 staining was also clearly seen, although with variable intensity, in the glomeruli of the CB parenchyma overlapping the expression of TH, which suggests that it was expressed by glomus cells (Figure, 1A). This immunohistochemical pattern was consistent in the human CB specimens studied (n=4) as well as in mouse CB (data not shown). In addition to the histological characterization of CB ACE2 protein expression, we used qPCR to analyze the levels of mRNA ACE2 expression in our cohort of human CBs (n=18). We found that the ACE2 mRNA levels are highly variable among all the CBs studied, without any clear correlation with donor age or gender (Figure, 1B). We did not see statistically significant differences in ACE2 mRNA levels associated with hypertension measured in a group of subjects (n=15) whose medical records cited evidence of blood pressure before death. A non-significant trend towards a higher level of ACE2 mRNA expression was seen in CBs from donors with blood type A with respect to type 0. However, these comparisons have no statistical value due to the small sample size.

The expression of ACE2 in the CB parenchyma suggests that $O_2$-sensing glomus cells could be potential targets for SARS-CoV-2 infection (Figure 1C). Growing evidence indicates that SARS-CoV-2 circulates in the blood and may infect, in addition to the lung epithelium, other tissues expressing ACE2, such as the olfactory https://mc.manuscriptcentral.com/function neuroepithelium, the cardiovascular system and the gastrointestinal tract, producing multiple and divergent functional alterations.[9] Thus, based on the high ACE2 expression found in human CB, it is plausible that the SARS-CoV-2 infection of chemosensory glomus cells could alter their ability to detect changes in arterial $O_2$ tension, resulting in unawareness of hypoxemia as occurs in case of “silent hypoxemia” observed in COVID-19 patients. Our data reveal a high individual variability of ACE2 expression in human CB tissue, which could explain why “silent hypoxemia” seems to appear randomly among COVID-19 patients. Regarding the mechanisms by which the potential SARS-CoV-2 infection could impair the CB hypoxic response, one possibility is that in an early phase the viral infection induces biochemical changes in glomus cells selectively altering their mitochondrial $O_2$-sensing mechanisms (Figure 1C). Indeed, it has been shown that 24 h after infection with SARS-CoV-2 the proteome of host human cells has already undergone extensive modulation affecting, among other proteins, enzymes of mitochondrial Krebs cycle and ETC subunits. Moreover, SARS-CoV-2 proteins synthesized in infected cells can interact with and hijack numerous host proteins, several of them normally expressed in mitochondrial matrix or assembled in mitochondrial ETC complexes.[10] Similar biochemical alterations in smooth muscle cells of small pulmonary arteries, which also have a mitochondrial-based $O_2$-sensing system, would result in the reduced hypoxic pulmonary vasoconstriction and large intrapulmonary shunt suggested to occur in COVID-19 patients.[1,3,4] In more advanced stages, CB SARS-CoV-2 infection could lead to inflammation and glomus cell death, thereby reducing the amount of chemosensitive elements in the CB and, henceforth, the ability to respond to hypoxemia (Figure 1C).

In conclusion, we suggest that SARS-CoV-2 CB infection could be the cause of, or contribute to, the “silent hypoxemia” observed in COVID-19 patients. Our proposal could be tested in autopsy studies of CB tissue obtained from COVID-19 patients and by experimental work using humanized ACE2-mouse transgenic models. In addition, if our hypothesis is correct, it would be necessary to study whether or not the damage produced by SARS-CoV-2 infection in the chemosensitive CB tissue is transient and to what extent it alters the regenerative potential of CB stem cells. If confirmed, our hypothesis would warrant the use of CB activators as respiratory stimulants in COVID-19 patients.[5] These drugs act downstream the mitochondrial $O_2$ sensor as they directly block $K^+$ channels in glomus cells.

https://mc.manuscriptcentral.com/function

### Conflicts of interest statement

None declared

### Funding

Experimental work in the authors' laboratories is supported by the Spanish Ministries of Science and Health (grants PID2019-106410RBI00, PID2019-105995RB-I00, and Red Temática de Investigación Cooperativa "Terapia Celular" RD16/0011/0025) and the European Research Council (ERC Advanced Grant PRJ201502629).

Figure 1. ACE2 expression and damage of carotid body $O_2$-sensing cells by SARS-CoV-2 infection. A. Histological sections (thickness 10 $\mu$m) of human carotid body showing ACE2 and TH staining with selective antibodies. DAPI was used to stain cell nuclei. v, blood vessel. B. Relative level of ACE2 mRNA expression in the carotid body tissue of the human subjects studied. RNA levels were calculated by qPCR with the $2^{-\Delta\Delta Ct}$ method using the average $\Delta$Ct value as a reference. The dashed line indicates the value of ACE2 mRNA expression used for normalization. C. Scheme illustrating the hypothetical consequences of infection of $O_2$-sensing glomus cells by SARS-CoV-2.

### References

1. Tobin MJ, Laghi F, Jubran A. Why COVID-19 silent hypoxemia is baffling to physicians. *Am J Respir Crit Care Med* 2020; 202: 356–360.
2. Couzin-Frankel J. The mystery of the pandemic's 'happy hypoxia'. *Science* 2020; 368: 455.
3. Archer SL, Sharp WW, Weir EK. Differentiating COVID-19 pneumonia from acute respiratory distress syndrome and high acute pulmonary edema. *Circulation* 2020; 142: 101–104.
4. Potus F, Mai V, Lebret M, et al. Novel insights on the pulmonary vascular consequences of COVID-19. *Am J Physiol Lung Cell Mol Physiol* 2020; 319: L277-L288.
5. Ortega-Sáenz P, López-Barneo J. Physiology of the carotid body: From molecules to disease. *Annu Rev Physiol* 2020; 82: 127–149.
6. Moreno-Domínguez A, Ortega-Sáenz P, Gao L, et al. Acute $O_2$ sensing through HIF2$\alpha$-dependent expression of atypical cytochrome oxidase subunits in arterial chemoreceptors. *Sci Signal* 2020; 13: eaay9452
7. Fung ML. Expressions of angiotensin and cytokine receptors in the paracrine signaling of the carotid body in hypoxia and sleep apnea. *Respir Physiol Neurobiol* 2015; 209: 6–12.
8. Hoffmann M, Kleine-Weber H, Schroeder S, et al. SARS-CoV-2 cell entry depends on ACE2 and TMPRSS2 and is blocked by a clinically proven protease inhibitor. *Cell* 2020; 181: 271–280.e8.
9. Li H, Liu L, Zhang D, et al. SARS-CoV-2 and viral sepsis: observations and hypotheses. *Lancet* 2020; 395**:** 1517–1520.
10. Gordon DE, Jang GM, Bouhaddou M, et al. A SARS-CoV-2 protein interaction map reveals targets for drug repurposing. *Nature* 2020; 583: 459–468.

https://mc.manuscriptcentral.com/function

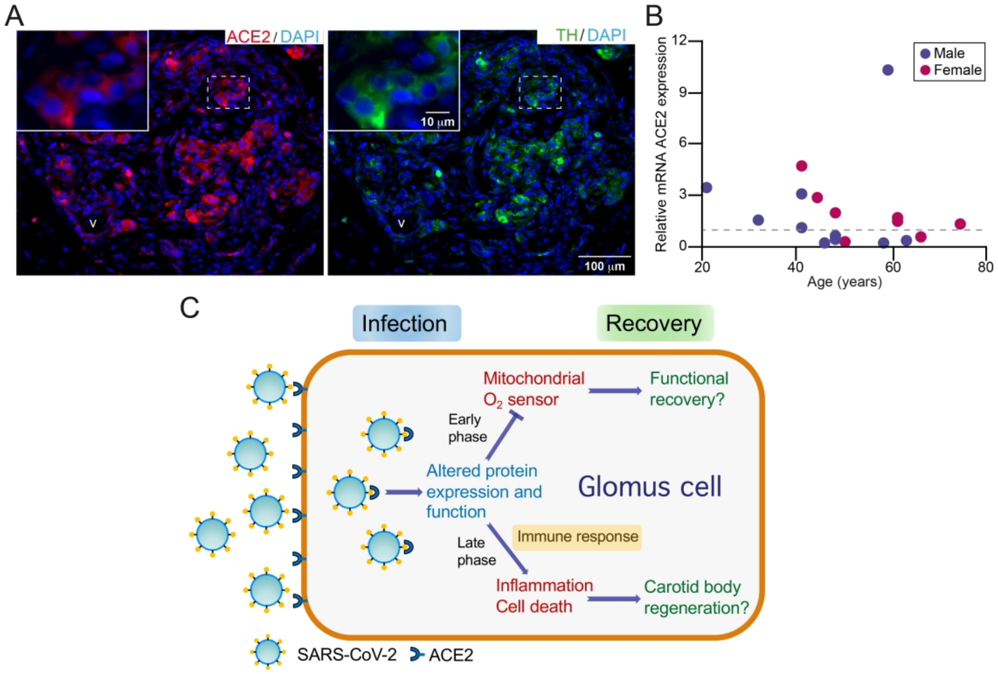

293x200mm (300 x 300 DPI)
